# Ebola Active Monitoring System for Travelers Returning from West Africa — Georgia, 2014–2015

**Published:** 2015-04-10

**Authors:** Mary Parham, Laura Edison, Karl Soetebier, Amanda Feldpausch, Audrey Kunkes, Wendy Smith, Taylor Guffey, Romana Fetherolf, Kathryn Sanlis, Julie Gabel, Alex Cowell, Cherie Drenzek

**Affiliations:** 1 Center for Surveillance, Epidemiology and Laboratory Services, CDC; 2 Georgia Department of Public Health; 3 Division of State and Local Readiness, CDC; 4 Office for State, Tribal, Local, and Territorial Support, CDC

The Ebola virus disease (Ebola) epidemic in West Africa has so far produced approximately 25,000 cases, more than 40 times the number in any previously documented Ebola outbreak (1). Because of the risk for imported disease from infected travelers, in October 2014 CDC recommended that all travelers to the United States from Ebola-affected countries receive enhanced entry screening and postarrival active monitoring for Ebola signs or symptoms until 21 days after their departure from an Ebola-affected country (2). The state of Georgia began its active monitoring program on October 25, 2014. The Georgia Department of Public Health (DPH) modified its existing, web-based electronic notifiable disease reporting system to create an Ebola Active Monitoring System (EAMS). DPH staff members developed EAMS from conceptualization to implementation in 6 days. In accordance with CDC recommendations, “low (but not zero) risk” travelers are required to report their daily health status to DPH, and the EAMS dashboard enables DPH epidemiologists to track symptoms and compliance with active monitoring. Through March 31, 2015, DPH monitored 1,070 travelers, and 699 (65%) used their EAMS traveler login instead of telephone or e-mail to report their health status. Medical evaluations were performed on 30 travelers, of whom three were tested for Ebola. EAMS has enabled two epidemiologists to monitor approximately 100 travelers daily,* and to rapidly respond to travelers reporting signs and symptoms of potential Ebola virus infection. Similar electronic tracking systems might be useful for other jurisdictions.

Active monitoring of travelers facilitates early detection of symptoms consistent with Ebola infection, rapid isolation of potential Ebola patients to prevent spread, and appropriate medical evaluation for prompt diagnosis. Active monitoring requires that travelers who are considered low (but not zero) risk (i.e., had been in Ebola-affected countries but had no reported contact with a person who was ill with Ebola) (3) report their health status to DPH once daily. The health status report includes their temperatures taken each morning and evening, whether they are experiencing any of a specified list of symptoms commonly associated with Ebola, and any other symptoms of illness. In Georgia, travelers categorized as having “some risk” for exposure (i.e., had contact with Ebola patients while wearing appropriate personal protective equipment) must be observed taking their temperatures each day by an epidemiologist via video direct active monitoring. “High-risk” travelers (i.e., had contact with an Ebola patient without adequate personal protective equipment) are quarantined upon arrival in their homes, or other location designated by DPH, if nonresidents, and also are observed via video connection for daily temperature checks. Active monitoring for Ebola can be labor intensive and costly (4). To reduce the burden of monitoring large numbers (>30 each week) of travelers arriving from Ebola-affected countries, DPH developed an automated system to assist with monitoring and data management.

## Development and Implementation of EAMS

DPH used the infrastructures of its State Electronic Notifiable Disease Surveillance System (SendSS) and its Public Health Information Portal to rapidly develop and deploy the web-based EAMS. Through close collaboration between DPH information technology development staff and epidemiologists responsible for initiating the active monitoring program, the core functions of EAMS were developed and deployed in 6 days. EAMS’s flexibility enables rapid updates for new data collection as surveillance needs are better understood.

EAMS consists of four components: 1) an online query capability designed to enable emergency departments to search EAMS by name and date of birth to quickly determine whether a patient is enrolled in active monitoring, 2) a traveler component that facilitates the online recording of daily symptom data, 3) a public health component that allows DPH epidemiologists to manage travelers throughout their active monitoring period, and 4) a reporting component that provides summary statistics, the capability to produce a line list of travelers, and a summary report to assist with weekly reporting to CDC. Epidemiologists in Georgia’s 18 health districts can log into the system to view and follow up with travelers in their own district; however, 14 districts have designated DPH to conduct monitoring.

The EAMS process begins when DPH epidemiologists create a record for each traveler from information obtained during entry screening and provided by CDC. The record for each traveler includes demographics, contact information, travel-related information, and the traveler’s risk categorization. Each record also contains a time-stamped progress notes section that facilitates communication among epidemiologists regarding individual records, including notes about noncompliance or symptomatic travelers. Once the record is created, an epidemiologist conducts a scripted telephone enrollment interview with the traveler to verify and complete information, and explain the system and monitoring requirements. The epidemiologist also provides the traveler with a legally binding Active Monitoring Agreement that explains the traveler’s responsibilities, instructions for reporting, and consequences of not reporting. After the enrollment process is completed, an EAMS system–generated e-mail is sent to the traveler that includes an individual username and password for accessing their EAMS account. Using their account, travelers can input their temperature and symptom checks each day into the secure system. Travelers can also report by telephone or e-mail if they prefer.

**FIGURE 1:**
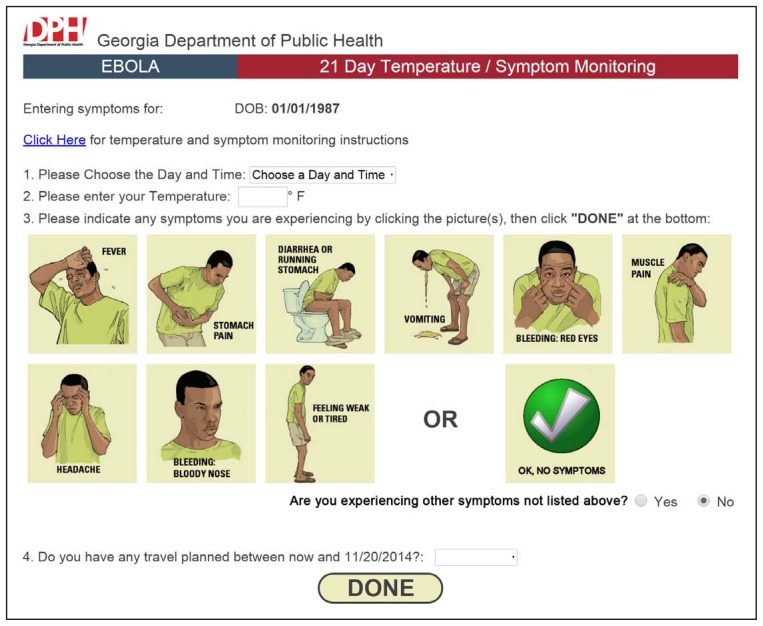
Ebola active monitoring system traveler symptom input screen for a fictitious traveler returning from West Africa — Georgia, 2014–2015

When reporting through EAMS, travelers log in, select the day and time, then enter their measured body temperatures. The system then prompts the traveler to indicate specific Ebola symptoms using pictorial selection boxes taken from the CDC-developed Ebola care kit (5), and offers a free-text box to enter other symptoms (Figure 1). Travelers also can enter details of any planned upcoming interstate or international travel during their monitoring period so that DPH can notify CDC and the receiving state.

**FIGURE 2:**
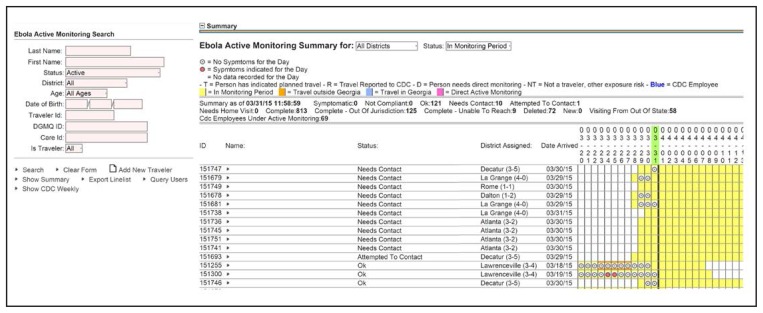
Ebola active monitoring system sample visual dashboard — Georgia, 2014–2015

Once travelers are enrolled, EAMS helps epidemiologists monitor travelers’ health and compliance with active monitoring through automated e-mail alerts and dashboards. Automated e-mail alerts notify epidemiologists when a traveler reports symptoms or a temperature >99.4° F (>37.2° C). Automated status updates enable the EAMS dashboard to clearly identify travelers who have not reported their temperature and symptom checks by a designated time so that epidemiologists can follow up and assure compliance (Figure 2). The visual dashboard displays the traveler’s name, the date of arrival in Georgia, the time remaining in the 21-day monitoring period, whether there are plans to travel to another state or country during the monitoring period, and whether this travel has been reported to CDC. Travelers who report fever or other signs or symptoms are labeled “symptomatic” and an email is sent to designated epidemiologists for follow-up. Travelers who do not report by 2 p.m., Eastern Time, are sent an automated email reminder. At 3 p.m., the status of travelers who have not reported becomes “noncompliant,” prompting epidemiologists to attempt contact. A status of “complete” is assigned at the end of travelers’ monitoring periods, and an automated e-mail informs them that they no longer need to report.

When symptoms are reported, the traveler is contacted by DPH. Low (but not zero) risk travelers who report mild symptoms (e.g., upper respiratory or gastrointestinal symptoms that don’t typically require seeing a clinician) are asked to self-isolate until symptoms subside. If more severe symptoms are reported, including any fever ≥100.4° F (≥38° C) with no other likely diagnosis, DPH epidemiologists coordinate with hospital preparedness personnel in DPH’s Emergency Preparedness Section to arrange medical evaluation at a designated hospital near the traveler’s current location that has the necessary isolation capabilities and willingness to screen potential Ebola cases. If a traveler needs urgent care or does not have private transportation, DPH will arrange transportation.

## Results of Active Monitoring

Active monitoring is conducted by two DPH epidemiologists each day. During October 25, 2014−March 31, 2015, DPH monitored 1,070 travelers (Table). The majority of travelers (65%) used the EAMS login system for one or more of their daily reports, and an estimated 85% reported on time each day to remain compliant. Thirty (2.8%) travelers received medical evaluations. Ebola testing was performed by real-time polymerase chain reaction on specimens from three travelers; all test results were negative. Among the 1,070 actively monitored travelers, 564 (53%) were CDC employees.

### Discussion

In October 2014, Ebola was diagnosed in a traveler from West Africa staying in Dallas (6). Thereafter, active monitoring was developed and implemented (2), enabling the timely detection of illness in travelers, which can facilitate early isolation of potential Ebola patients to prevent the spread of disease, appropriate medical evaluation, and early detection and management of Ebola. EAMS makes it possible for two epidemiologists to monitor approximately 100 travelers each day. It achieves this 1) by allowing travelers to report their own monitoring information via computer or web-enabled mobile telephone, 2) by providing a summary dashboard to allow epidemiologists to quickly assess the status of travelers, and 3) by sending automated e-mail alerts to epidemiologists when symptoms are reported.

Because monitoring must occur every day, including on weekends and holidays, having a web-based system that is accessible from any computer helped foster acceptability among monitoring personnel. The simplicity of EAMS enables travelers to enter their own information if they choose and allows for many travelers to be managed by few epidemiologists. Ease of use for the travelers has resulted in a high level of acceptability, with 65% of travelers choosing to use EAMS direct login over sending e-mails or telephone messages. Most importantly, the instant e-mail alert of reported symptoms to DPH epidemiologists provides timely notification of illness among travelers.

Monitoring for Ebola is necessary to detect and isolate cases early, facilitate medical evaluation, and prevent its spread. Georgia, with its large number of travelers and limited number of DPH epidemiologists, needed an efficient system to ensure the success of its monitoring program. Including DPH’s information technology staff as members of Georgia’s Ebola response team was crucial to Georgia’s ability to develop this flexible online module in 6 days. Similar systems might be useful for other jurisdictions and might potentially reduce the cost of monitoring (4). EAMS also might serve as a model for meeting the surveillance needs of other public health programs in a timely manner.

What is already known on this topic?

Because Ebola can only be transmitted through close contact with a person who has developed symptoms, close monitoring of persons with potential exposure facilitates early identification of suspected cases, appropriate medical evaluation, and rapid isolation to prevent further spread.

What is added by this report?

Modifying and leveraging the existing infrastructure of the current Georgia State Electronic Notifiable Disease Surveillance System has provided the flexibility for two staff members to efficiently and effectively monitor approximately 100 travelers from Ebola-affected countries on a daily basis.

What are the implications for public health practice?

Simple electronic tools can be adapted or developed for active monitoring and make data easily accessible to epidemiologists. Such systems also can enable travelers being monitored to take an active role in their own reporting. The system has been instrumental in the successful monitoring of Georgia’s travelers from Ebola-affected countries, and similar systems might be useful for other jurisdictions.

## Figures and Tables

**TABLE:** Number of travelers from Ebola-affected countries ( N = 1,070) actively monitored for signs and symptoms of Ebola, by selected characteristics — Georgia, October 25, 2014-March 31, 2015

Characteristic	No.	(%)
Total monitored	1,070	(100)
Average no. monitored per day*	114	—
Completed monitoring	957†	(89)
Reported using EAMS log-in§	699	(65)
CDC employees monitored	564	(53)
Medical evaluation performed	30	(2.8)
Tested for Ebola¶	3	(0.2)

**Abbreviations:** EAMS = Ebola Active Monitoring System.

* During December 2014–March 2015.

† As of March 31, 2015; a total of 113 other travelers were still being actively monitored.

§ Travelers logged temperature and symptom reports directly into EAMS for at least one daily report.

¶ Tested by real-time polymerase chain reaction at an Ebola reference laboratory.

## Footnotes

* Maintaining a precise count is difficult because persons move in and out of EAMS monitoring every day. Since December, however, the daily average has been 114, with a range of 81–153 per day.

## References

[1] CDC (2014). Ebola (Ebola virus disease) Outbreaks chronology.

[2] CDC (2014). Ebola (Ebola virus disease) Risk of exposure Interim US guidance for monitoring and movement of persons with potential Ebola virus exposure.

[3] CDC (2014). Ebola (Ebola virus disease) Risk of exposure Epidemiologic risk factors to consider when evaluating a person for exposure to Ebola virus.

[4] Yacisin K, Balter S, Fine A (2015). Ebola virus disease in a humanitarian aid worker—New York City, October 2014. MMWR Morb Mortal Wkly Rep.

[5] CDC (2014). Ebola (Ebola virus disease) Travelers Ebola care kit.

[6] Chervalier MS, Chung W, Smith J (2014). Ebola virus disease cluster in the United States—Dallas County, Texas, 2014. MMWR Morb Mortal Wkly Rep.

